# FGF23 and Fetuin-A Interaction and Mesenchymal Osteogenic Transformation

**DOI:** 10.3390/ijms20040915

**Published:** 2019-02-20

**Authors:** Deborah Mattinzoli, Masami Ikehata, Koji Tsugawa, Carlo M. Alfieri, Mario Barilani, Lorenza Lazzari, Paola Andreetta, Francesca M. Elli, Giovanna Mantovani, Piergiorgio Messa

**Affiliations:** 1 Renal Research Laboratory Fondazione IRCCS Ca’ Granda Ospedale Maggiore Policlinico, 20122 Milan, Italy; deborah.mattinzoli@policlinico.mi.it (D.M.); masami.ikehata@policlinico.mi.it (M.I.); kojituga@outlook.com (K.T.); carlo.alfieri@policlinico.mi.it (C.M.A.); poline.92.pa@gmail.com (P.A.); 2 Unit of Nephrology, Dialysis and Renal transplant Fondazione IRCCS Ca’ Granda Ospedale Maggiore Policlinico, 20122 Milan, Italy; 3 EPIGET LAB, Department of Clinical Sciences and Community Health, University of Milan, 20122 Milan, Italy; mario.barilani@policlinico.mi.it; 4 Department of Transfusion Medicine and Hematology, Cell Factory, Regenerative medicine laboratory, Fondazione IRCCS Ca’ Granda Ospedale Maggiore Policlinico, 20122 Milan, Italy; lorenza.lazzari@policlinico.mi.it; 5 Department of Clinical Sciences and Community Health, Endocrinology Unit, University of Milan, 20122 Milan, Italy; francesca.elli@unimi.it (F.M.E.); giovanna.mantovani@policlinico.mi.it (G.M.); 6 Unit of Endocrinology and Metabolic Diseases, Fondazione IRCCS Ca’ Granda Ospedale Maggiore Policlinico, 20122 Milan, Italy; 7 Department of Clinical Sciences and Community Health, University of Milan, 20122 Milan, Italy

**Keywords:** FGF23, Fetuin-A promoter, mesenchymal cell, osteogenesis, chronic kidney disease

## Abstract

Recently, we found a strict bone association between Fibroblast growth factor 23 (FGF23) and Fetuin-A, both involved in cardiovascular and mineral bone disorders. In this study, an uninvestigated bone marrow positivity for both was found. Though the role of exogenous FGF23 on mesenchymal cells (MSCs) was reported, no information is as yet available on the possible production of this hormone by MSCs. To further analyze these uninvestigated aspects, we studied human primary cells and mouse and human cell lines by means of immunostaining, qRT-PCR, enzyme linked immunosorbent assays, chromatin immunoprecipitation, transfection, and a streamlined approach for the FGF23–Fetuin-A interaction called Duolink proximity ligation assay. Mesenchymal cells produce but do not secrete FGF23 and its expression increases during osteo-differentiation. Fibroblast growth factor 23 is also involved in the regulation of Fetuin-A by binding directly to the Fetuin-A promoter and then activating its transcription. Both FGF23 overexpression and addition induced an upregulation of Fetuin-A in the absence of osteo-inducer factors. Fibroblast growth factor 23 and Fetuin-A promoter were increased by osteo-inducer factors with this effect being abolished after FGF23 silencing. In conclusion, both FGF23 and Fetuin-A are present and strictly linked to each other in MSCs with FGF23 driving Fetuin-A production. This mechanism suggests a role for these two proteins in the osteoblast differentiation.

## 1. Introduction

Over the last decade, research has increasingly focused on mesenchymal cells (MSCs) as potentially useful tools in medicine. MSCs have manifold activities, including the capacity for migration to injury sites for tissue regeneration, the self-renewal aptitude, and both the high ability for cell differentiation and modulatory regulation of the inflammatory system [1,2,3,4,5]. 

Mesenchymal cells are the progenitor of myoblasts, chondrocytes, adipocytes, and osteoprogenitors, and their lineage predestination are defined by different transcription factors. Runt-related transcription factor 2 (RUNX2) and osterix are the osteoblast (OB) transcription factors that, in addition to proto-oncogene protein Wnt signaling, regulate the osteo-differentiation process [6]. Different regulatory genes and molecular markers characterize different stages of the osteo-differentiation process. Osteoblasts are defined by their ability to synthesize mineralized matrix proteins such as collagen Type I (COLIαI), osteocalcin (BGLAP), and alkaline phosphatase activity (ALP) by their responsiveness to specific hormones and growth factors and their position in the bone tissue [7,8]. Osteocytes (OS), derived from OBs, are trapped in the bone matrix and characterized by several long processes creating a complex network with the other bone cells. Osteocytes play the role of the orchestrator of the bone remodeling events, organizing both resorption and formation, representing the major cells responding to mechanical stimuli [9,10,11,12]. 

It well known that many growth factors are involved in the differentiation process of MSCs towards different lineages including osteoblastogenesis [13,14,15,16,17,18]. Fibroblast growth factor 23 (FGF23) is a 32 kD protein identified as a hormonal substance mainly secreted by the OS. Acting through its four receptors and through the transmembrane protein α-Klotho, FGF23 inhibits in the kidney the phosphate reabsorption by decreasing Na-dependent co-transporters and suppresses the circulating 1,25(OH)_2_D levels inhibiting Cyp27b1 (which converts 25(OH)D to 1,25(OH)_2_D) and stimulating the catabolism of 1,25(OH)_2_D by activation of the 24-hydroxylase (Cyp24) [19]. Fibroblast growth factor 23 plays a key role in skeletal development, being also involved in the metabolism of phosphate, calcium, parathyroid hormone, and vitamin D, all factors interconnected with the complex network related to mineral metabolism [20]. 

The role of FGF23 on both MSCs and premature OBs is, as yet, not completely clarified: a study reported that high dosage of FGF23 in pre-OB MC3T3-E1 cell line stimulated the proliferation and inhibited the mineralization of MSCs [21], while other authors reported that concentrations 1000-fold lowered directed the MSCs toward OB differentiation [22]. These contradictory results might be at least dependent on the different FGF23 concentrations and cell differentiation stages. Furthermore, it has not yet been reported on a possible endogenous production of FGF23 by MSCs themselves. 

In fact, in one of our previous papers, we found an unexpected positivity for FGF23 expression in mice bone marrow tissue [23,24].

The first aim of the present study was to explore if MSCs produce FGF23 and if it could be involved in the osteo-differentiation process.

In our previous study, we also found that OSs produce Fetuin-A and that its production is highly regulated by FGF23, with a strict relationship between the two proteins both in the nucleus and in the cytoplasm. This strict inter-relationship between these two proteins was also described in the circulation and in liver cells [25]. Fetuin-A has long been known to be involved in multifunctional activities, such as vascular calcification, bone mineral regulation, anti-inflammatory role, cell migration, etc. Therefore, Fetuin-A protein expression could represent a protection against several diseases: mineral bone disorder (MBD), cardiovascular disease (CVD), chronic kidney disease (CKD), and cancer [26,27,28]. 

Since no study has reported on a possible production of Fetuin-A by MSCs, the second aim of our investigation was to explore also for this possibility. 

## 2. Results

### 2.1. FGF23 and Fetuin-A Expression in Tissue and Cells Mice Bone Marrow 

**Figure 1:**
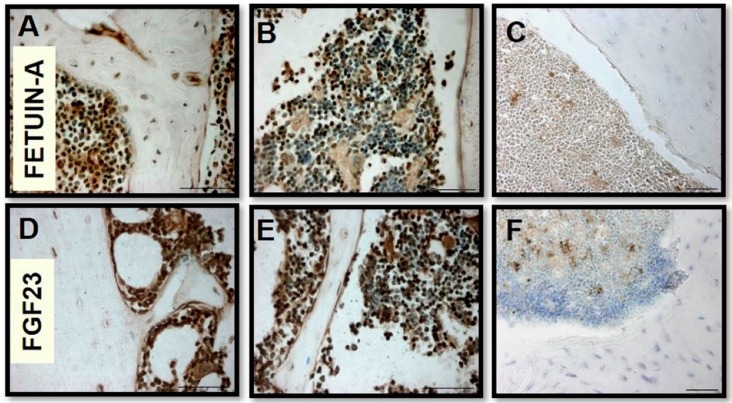
Immunostaining of Fetuin-A (**A**,**B**) and Fibroblast growth factor 23 (FGF23) (**D**,**E**) in mice bone marrow tissues. Negative control, respectively, (**C**) and (**F**). Scale bar = 50 μm.

**Figure 2:**
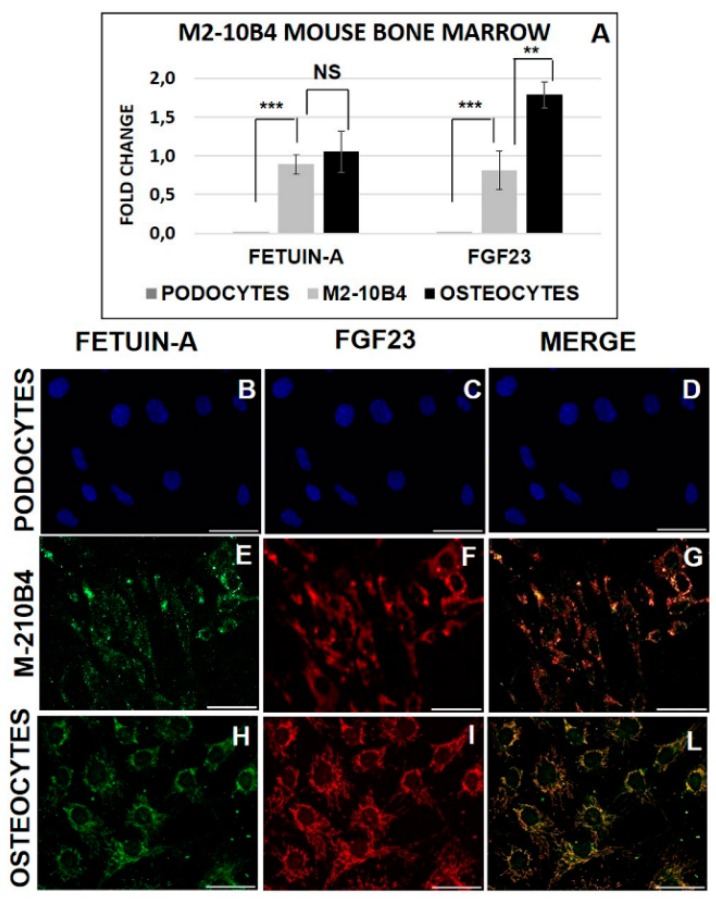
qRT-PCR of Fetuin-A and FGF23 mRNA expression in podocytes (PODO) (control negative), M2-10B4 bone marrow cells, and osteocytes (OS) (control positive) (**A**). Fetuin-A (**B**,**E**,**H**), FGF23 (**C**,**F**,**I**), and merge (**D**,**G**,**L**) expression visualized by FITC and rodamine, respectively in PODO, M2-10B4, and OS. DAPI staining is deliberately shown to demonstrate the cells’ presence (**B**,**C**,**D**): ** *p* < 0.01, *** *p* < 0.001. Scale bar = 50 μm.

Fetuin-A (Figure 1A,B) and FGF23 (Figure 1D,E) evaluated by IHC were highly expressed in bone MSCs extracted from tibial tissue of Balb/c mice, as shown by the high positivity of MSCs for diaminobenzidine compared to the negative control (Figure 1C,F). Since it is known that DAB is not specifically bound by blood cells, in order to confirm the real BMC positivity of both FGF23 and Fetuin-A, we tested the mRNA expression of both markers in M2-10B4, compared with OS (control positive) and PODO (control negative). We found that both MSCs and OS highly express both FGF23 and Fetuin-A, though FGF23 expression was lower in MSCs than in OS, at variance with PODO which confirmed their negativity for the expression of both markers (Figure 2A). The IF of Fetuin-A (Figure 2B,E,H), FGF23 (Figure 2C,F,I) and their MERGE (Figure 2D,G,L), tested respectively in PODO, M2-10B4, and OS cells, confirmed the presence of both proteins in M2-10B4 and OS, where they were also strictly co-localized while no expression of either proteins was confirmed in PODO.

### 2.2. FGF23 and Fetuin-A Expression in Primary and Human MSCs 

**Figure 3:**
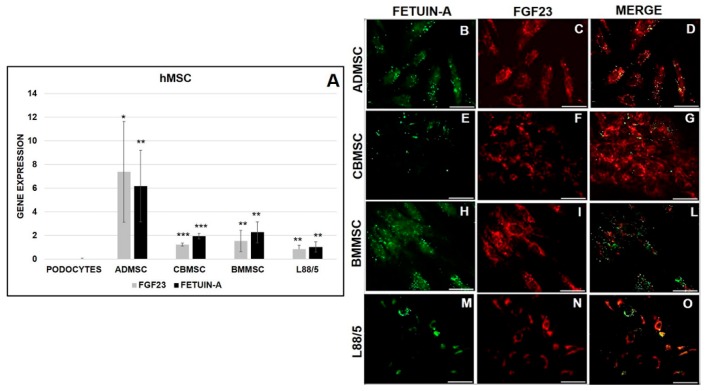
qRT-PCR of Fetuin-A and FGF23 mRNA expression in PODO (control negative), ADMSC, CBMSC, BMMSC, and L88/5 cell line (**A**). IF of Fetuin-A (**B**,**E**,**H**,**M**), FGF23 (**C**,**F**,**I**,**N**), and MERGE (**D**,**G**,**L**,**O**) in the same human MSCs. Asterisks indicate significant differences versus PODO: * *p* < 0.05, ** *p* < 0.01, *** *p* < 0.001. Scale bar = 50 μm.

For completeness, we also examined the real quantity of Fetuin-A and FGF23 mRNA expression in the three different human primary MSCs, namely ADMSC, CBMSC, and BMMSC, and the L88/5 cell line compared to PODO (control negative). The primary cells ADMSC, CBMSC, BMMSC and the cell line L88/5 expressed a significant amount of both markers (Figure 3A). The IF evidenced the protein expression of Fetuin-A (Figure 3B,E,H,M) and FGF23 (Figure 3C,F,I,N) confirming a partial co-localization with a part of Fetuin-A not colocalized (Figure 3D,G,L,O). 

### 2.3. FGF23 Release

**Figure 4:**
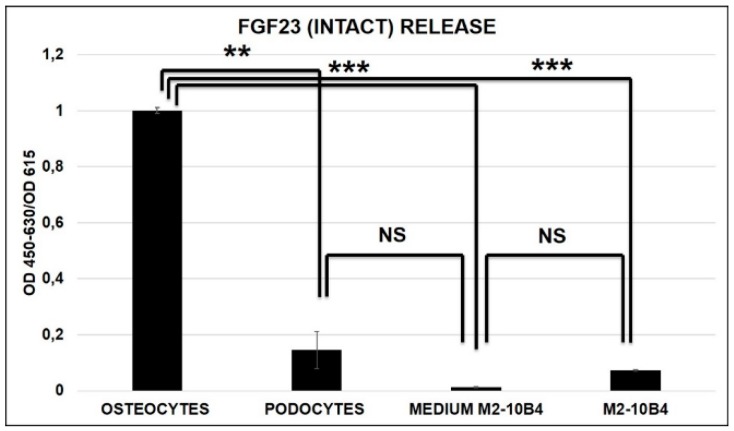
Cell cultured medium harvested from OS, PODO, M2-10B4, and basal medium for measurement of intact FGF23 release assessed by ELISA: ** *p* < 0.01, *** *p* < 0.001.

At variance with OS (control positive), no FGF23 release was detected in M2-10B4 without osteo-induction and PODO as well as in M2-10B4 media (control negative) (Figure 4).

### 2.4. Effect of Osteogenic Differentiation on FGF23 and Fetuin-A Expression 

During the natural MSC growth, we observed a reduction of both FGF23 and Fetuin-A expression in M2-10B4, but not in L88/5 (Figures S3A and S4A). No FGF23 release (Figures S3B and S4B) or FGF23/Fetuin-A interaction (Figures S3C and S4C) occurred in either cell lines during their growth from T0 to T15. 

**Figure 5:**
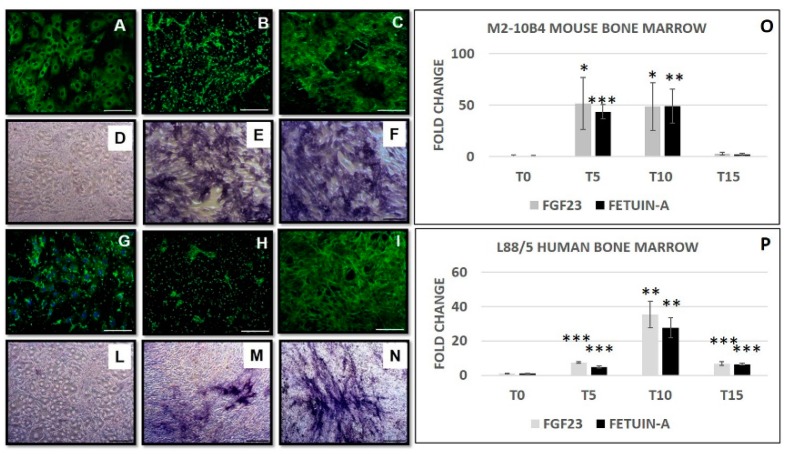
COLIαI and ALP staining in mouse M2-10B4 (**A**–**C**)/(**D**–**F**) and in human L88/5 (**G**–**I**)/(**L**–**N**) bone marrow cells from T0 to T10 days from the osteogenic induction. qRT-PCR of Fetuin-A mRNA expression and FGF23 in M2-10B4 (**O**) and L88/5 bone marrow cells (**P**) from T0 to T15 days from the osteo-differentiation. Asterisks indicate significant differences versus M2-10B4 and L88/5 T0: * *p* < 0.05, ** *p* < 0.01, *** *p* < 0.001. Scale bar = 100 μm (**A**–**C**/**G**–**I**), 200 μm (**D**–**F**/**L**–**N**).

During the osteo-induction, both M2-10B4 (Figure 5A–C and D–F) and L88/5 (Figure 5G–I and L–N) cells showed an increase in COLIαI and ALP staining. At the same time, FGF23 and Fetuin-A mRNA expression significantly increased from T0 to T10 and then decreased from T10 to T15 during osteo-induction in both cell lines (Figure 5O,P). At T10 we obtained the complete OB transformation evaluated in M2-10B4 cells by the BGLAP increase (Figure S5), as indicated in the Takara osteoblast inducer kit data sheet. Collagen deposition was also confirmed by Picrosirius red staining on both M2-10B4 and L88/5 (Figure S6 A–C/D–F). The levels of both FGF23 and Fetuin-A were also examined in the adipocytes and chondrocytes differentiation process. Fibroblast growth factor 23 expression was non-significant in both adipocytes and chondrocytes at T21 compared to bone marrow at T0. Fetuin-A was upregulated only in the adipocytes but not in the chondrocytes (Table S3). 

### 2.5. FGF23 Release during MSCs Transformation in OB

**Figure 6:**
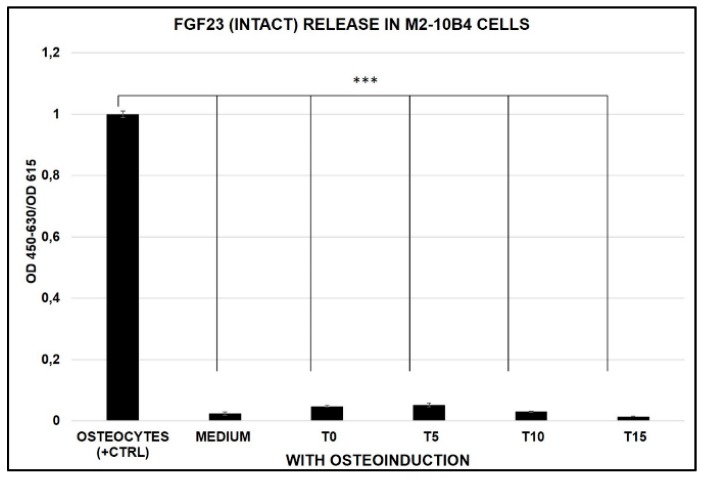
Intact FGF23 release in cell cultured medium harvested from OS (control positive) and M2-10B4 during the osteogenic induction (T0–T15): *** *p* < 0.001.

We then explored whether any release of FGF23 occurs during the osteo-inductive treatment. As in conditions without osteo-induction, no release of FGF23 was evident from T0 to T15 compared to OS (control positive) (Figure 6).

### 2.6. Interaction between FGF23 and Fetuin-A

#### 2.6.1. Fetuin-A and FGF23 Interaction in Mouse, in Primary Human Cells and Tibial Mouse Tissue 

**Figure 7:**
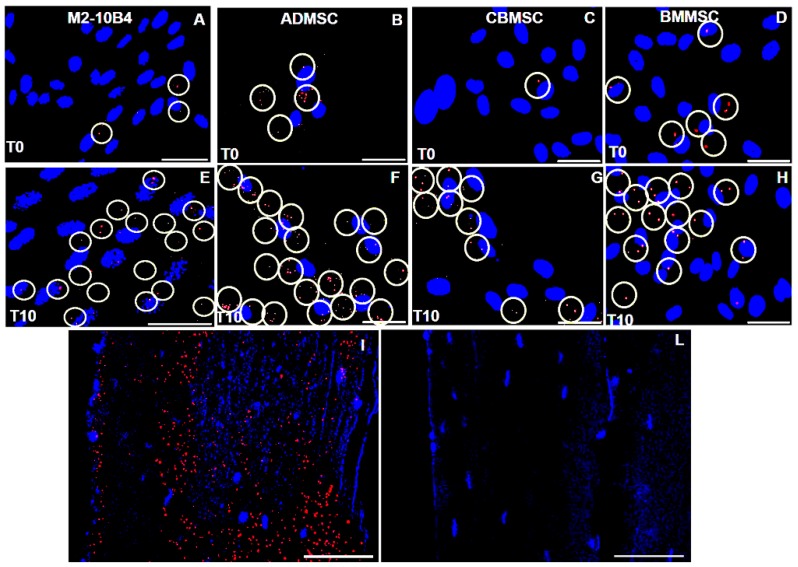
The white circles highlight the cell localization of Fetuin-A/FGF23 interaction detected by a Duolink in situ experiment in M2-10B4 (**A**,**E**), ADMSC (**B**,**F**), CBMSC (**C**,**G**), and BMMSC (**D**–**H**) during the osteogenic induction from T0 to 10 days. FGF23 /Fetuin-A interaction in mice tibial bone tissue detected by Duolink in red, and DAPI for the cells localization in blue (**I**), control negative (**L**). Scale bar = 50 μm.

We then explored if FGF23/Fetuin-A co-localization in MSCs is characterized by a strict interaction either in conditions without osteo-induction or during osteogenic differentiation. A strict interaction was evident both in M2-10B4 (Figure 7A) and different primary human MSCs (Figure 7B–D), moreover this interaction considerably increased up to T10 (Figure 7E–H). The same degree of a strict FGF23/Fetuin-A co-localization was evident also in tibial mice tissue. A positivity was observed to the border between bone marrow and pre-OB and in the OS (Figure 7I), in Figure 7L the relative control negative. 

#### 2.6.2. Nuclear Biochemical Interaction between FGF23 and Fetuin-A promoter

**Figure 8:**
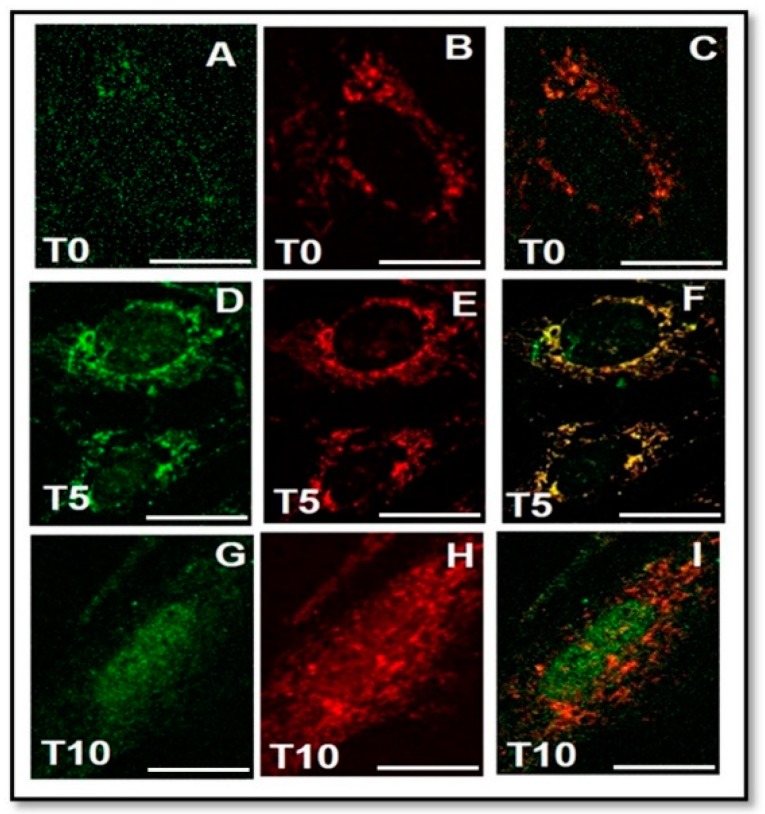
Immunofluorescence of Fetuin-A (**A**,**D**,**G**), FGF23 (**B**,**E**,**H**) and MERGE (**C**,**F**,**I**), respectively, at T0, T5, and T10 in M2-10B4. Scale bar = 20 μm.

Since we observed an evident co-localization of FGF23/Fetuin-A also in the nucleus of MSCs, (Figure 8) and given that such a co-localization was observed also in OS in our previous study [23,24], we performed a CHIP with FGF23, followed by a PCR for the identification of the Fetuin-A promoter, both in OS and M2-10B4 cells. Moreover, we decided to use primers chosen in different parts of the Fetuin-A promoter for the validation of the experiments.

**Figure 9:**
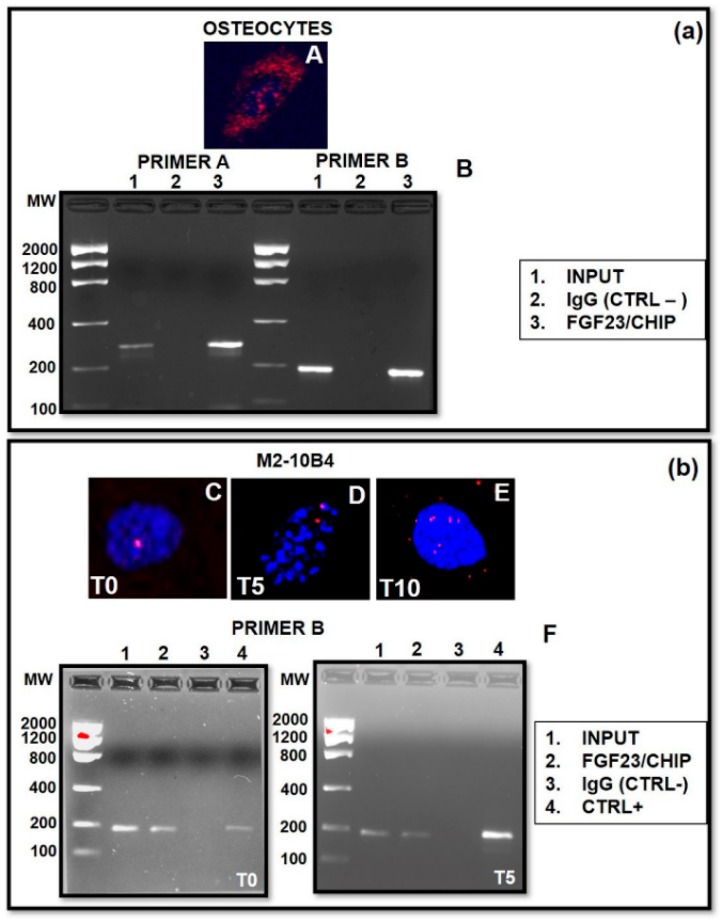
Nuclear localization of Fetuin-A/FGF23 interaction detected by the Duolink in situ experiment in OS T5 (**A**). Semi-quantitative PCR of Fetuin-A promoter on fragment immunoprecipitated by FGF23 of the OS Chromatin (**B**). Primer A and primer B represent 2 distinct types of primers on the same Fetuin-A promoter to validate the data. (**b**) Nuclear localization of Fetuin-A/FGF23 interaction detected by the Duolink in situ experiment in M2-10B4 at T0–T10 (**C**–**E**). Semi-quantitative PCR of Fetuin-A promoter on the fragment immunoprecipitated by FGF23 of the OS Chromatin (**F**). In both experiments the false positivity was excluded by the control negative, while the correctness of the PCR was confirmed by the control positive and the input.

The nuclear interaction in the OS was not only reconfirmed by Duolink (Figure 9A) but also by the bands respectively at 279 bp and 189 bp corresponding to Fetuin-A promoter sequences (Figure 9B). 

Similarly, a nuclear interaction was evident also in M2-10B4, with its intensity increasing from T0–T10 (Figure 9C–E). We also observed a signal positivity at 189 bp at both T0 and T5, indicating that the fragment immunoprecipitated by FGF23 likely corresponds to the Fetuin-A promoter (Figure 9F). 

#### 2.6.3. FGF23 Overexpression and Addition on both FGF23 Expression and Fetuin-A Promoter Activation

**Figure 10:**
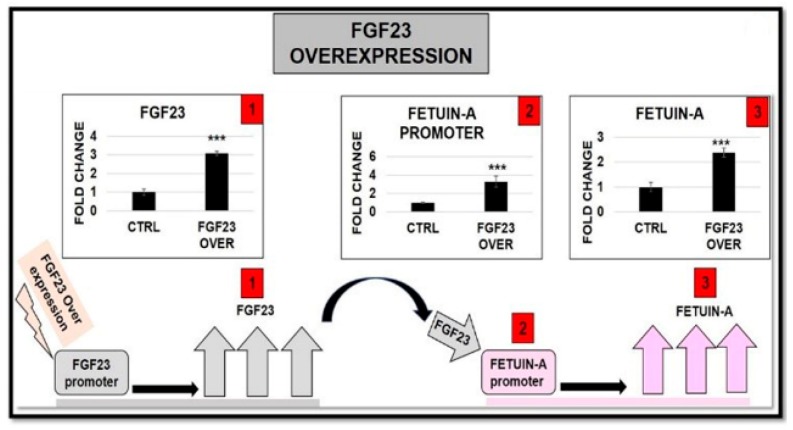
qRT-PCR of FGF23 (1), Fetuin-A promoter (2), and Fetuin-A 24 h (3) after FGF23 overexpression: *** *p* < 0.001.

To deepen the influence of FGF23 on Fetuin-A, we performed an FGF23 overexpression on M2-10B4 without osteo-induction. The expected increased expression of FGF23 was associated with a significant increase of Fetuin-A promoter activity (Figure 10). 

Thereafter we explored the effects of the FGF23 addition at different concentrations (400, 800, 1600 pg/mL) and at different times of osteo-differentiation (T0, T5, T7).

**Figure 11:**
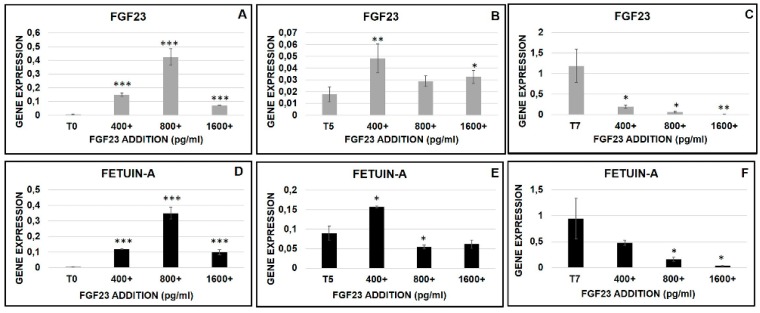
qRT-PCR of FGF23 (**A**–**C**) and Fetuin-A (**D**–**F**) 24 h after 400, 800, 1600 pg/ml of FGF23 addition at T0, T5, and T7 during the osteo-induction (**A**). Asterisks indicate significant differences versus M2-10B4 T0, T5, and T7 without induction: * *p* < 0.05, ** *p* < 0.01, *** *p* < 0.001.

The addition of FGF23 induced an increased expression of both FGF23 and Fetuin-A mRNAs, at T0 and T5, with an inhibitory effect on both at T7. Furthermore, the kinetics of dose-dependent stimulation at T0 and T5 was different, since at T0 a dose-dependent up- regulation of both mRNAs was observed at 400 and 800pg/mL, followed by a decline at 1600 pg/mL (Figure 11A,D). At T5, FGF23 mRNA was maximally upregulated at 400 pg/mL, declining progressively with further increased FGF23 doses, while Fetuin-A mRNA was stimulated at 400 pg/mL but inhibited at higher FGF23 levels (Figure 11B,E). At T7, the addition of FGF23 was followed by a down regulation of both mRNAs at all concentrations (Figure 11C,F). 

#### 2.6.4. FGF23 Influence on Osteo-Differentiation Markers during the OB Transformation

**Figure 12:**
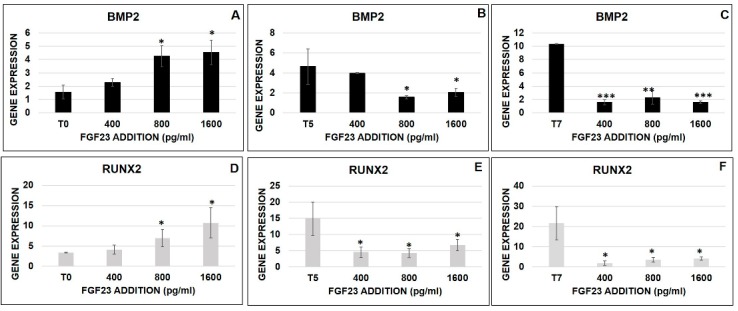
qRT-PCR of BMP2 (**A**,**B**,**C**) and RUNX2 (**D**,**E**,**F**) 24 h after 400, 800, 1600 pg/mL of FGF23 addition at T0, T5, and T7 during the osteo-induction (**A**). Asterisks indicate significant differences versus M2-10B4 T0, T5, and T7 without induction: * *p* < 0.05, ** *p* < 0.01, *** *p* < 0.001.

To investigate the effect of FGF23 addition on osteo-differentiation, we analyzed the BMP2 and RUNX2 mRNA expression at different concentrations (400, 800, 1600 pg/mL) and at different times of osteo-differentiation (T0, T5, T7). In these experiments, the osteo-induction in MSCs, in the absence of FGF23 addition, induced an increase of both BMP2 and RUNX2 expression from T0 to T7 (Figure 12A–F). When FGF23 was added at T0, a stepwise stimulation of both proteins was evident, starting to be significant at 800 pg/mL (Figure 12A,D). At variance, the addition of 400 pg/mL of FGF23 at T5 stops inducing the increase of both proteins. The subsequent additions (800 and 1600 pg/mL) at T5 and any addition at T7 reduced the expression of BMP2 and RUNX2 (Figure 12B–F). 

#### 2.6.5. FGF23 Influence on Fetuin-A Promoter Expression during the OB Transformation 

**Figure 13:**
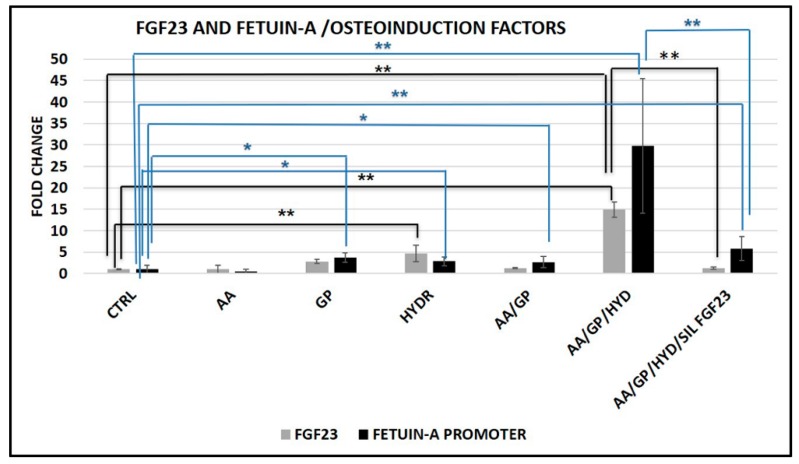
qRT-PCR of FGF23 and Fetuin-A promoter 5 days after AA, GP, HYD, AA/GP, and AA/GP+ HYD addition. The last condition represented all conditions added with FGF23 silencing for 24 h. * *p* < 0.05, ** *p* < 0.01.

For completing our knowledge on the osteo-differentiation process, we also explored the possible role of each single factor used in our model. Singularly adding each factor, we found that only GP and HYD increased Fetuin-A promoter, with no significant effect of AA. We also observed a synergistic effect of AA/GP and even more of AA/GP/HYD together on Fetuin-A promoter activity (Figure 13).

Neither AA and GP alone or AA/GP together increased FGF23 expression; on the contrary, HYD alone and even more when associated with AA/GP strongly stimulates FGF23 expression. 

The maximum stimulatory effects obtained with all synergic reagents on FGF23 and Fetuin-A promoter activity were significantly downsized after FGF23 silencing (Figure 13).

## 3. Discussion

In our previous studies [23,24] we already described a tight control of FGF23 on Fetuin-A production in the OS, with some aspects remaining to be explored. 

The main aim of the present study was to confirm the presence of both FGF23 and Fetuin-A in the bone marrow and their possible relationships both at the cytoplasmic and nuclear level. All experiments were conducted on mouse cell line, and the addition of some data on human primary cells and human cell line confirmed our results. 

From the analysis of mice tibial bone marrow, a strong positivity was evident for both proteins. However, since endogenous peroxidase is present in most blood cells, endogenous peroxidase could affect the reaction of DAB, despite using a blocking reagent, giving a false positivity for both proteins [29]. To gain more certainty of the real positivity for both markers in MSCs bone marrow cells, we compared a bone marrow cell line (M2-10B4) with the OS (control positive) and the PODO (control negative).

Podocytes are particular glomerular cells equipped with regularly interconnected foot processes involved in the glomerular filtration selectivity [30]. The choice to use PODO as a negative control was linked to our laboratory background since we have been involved in the PODO study for many years [31,32,33], and therefore are confident regarding its negativity for FGF23 and Fetuin-A expression. In fact, in our previous study exploring FGF23 and Fetuin-A in both renal tubular and glomerular cells, we did not find any FGF23 expression in the glomerulus [25]. In addition, we decided to use OS as a positive control since, as is already well known in the literature, OS are recognized as the main source of circulating (hormonal) FGF23.

In fact, FGF23 expression was lower in MSCs than in OS, while the expression of Fetuin-A was almost the same in OS as in M2-10B4. No quantifiable amount of either FGF23 and Fetuin-A was found in PODO. Though interactions between circulating FGF23 and Fetuin-A with MSCs have already been reported [34,35,36]. To the best of our knowledge, this is the first time that FGF23 and Fetuin-A have demonstrated being expressed also in MSCs. Another important finding was that FGF23 and Fetuin-A were clearly co-localized both in the cytoplasm and nucleus of MSCs. Given the MSCs variability linked to their multipotentiality, to the culture method susceptibility, to the tissue origin, and to the donor genotype, we also explored the expression of both molecules in the three principal human sources (i.e., bone marrow, adipose tissue, and umbilical cord blood) [37,38,39]. In all the primary MSCs cells and in the human L88/5 lineage, despite the expected variability due to the different source, both FGF23 and Fetuin-A were significantly increased as compared with PODO. Also, in this case, even if at a lesser extent, IF showed a partial and variable co-localization of both molecules highlighting a punctuated Fetuin-A already observed in our previous study [25]. To better understand the meaning of FGF23 production in MSCs, we looked for a possible release. Despite the FGF23 expression, no release was observed by M2-10B4. A further question is related to the time dependent behavior of the production of the two proteins during the osteo-differentiation process. First, we explored whether any change of FGF23 and Fetuin-A expression and interaction occurred during the natural cell growth, focusing on M2-10B4 mouse and L88/5 human bone marrow cells line, due to their lower variability as compared with primary cells. In fact, we observed that both proteins did not increase in both M2-10B4 and L88/5 cells during their growth without any stimulation. Then, we explored the behavior of both proteins in other differentiation lineages (adipocytes and chondrocytes) at T21. In both cases only adipocytes significantly expressed Fetuin-A, as expected, since Fetuin-A is mainly secreted by both liver and adipocytes [40,41,42]. Finally, we moved to explore the time-dependent behavior during the osteo-differentiation process in the same cell lines. Testing the correct osteo-differentiation by COL1 and ALP staining [43], we observed that both FGF23 and Fetuin-A expression increased from T0 to T10 days decreasing thereafter (T15). As reported by the Takara kit and confirmed by BGLAP expression, T10 is the time when the MSCs turn into OB lineage, though as yet at an immature stage, while at T15 the cells gain a full OB characteristic. 

This behavior confirms our previous results where FGF23 and Fetuin-A were highly expressed in the pre-OB, significantly downsized in the mature OB, and eventually achieving maximal expression with further maturation into OS [23,24]. However, no FGF23 release was observed during the OB transformation, while OS are well recognized to release this protein. These results suggest that OB might represent a turning point in the osteo-differentiation process of MSCs toward the final form of OS, passing from cell types able to produce and not secrete FGF23 (perhaps a autocrine/paracrine function) to a cell type specialized in FGF23 secretory property (endocrine function). It is worth noting that the behavior of FGF23 was paralleled by that of Fetuin-A over the whole osteo-differentiation process. We then decided to investigate if FGF23 was linked to Fetuin-A. By Duolink test, we observed a strict co-localization of the two proteins, both in the cytoplasm and nucleus of both M2-10B4 and primary cells, with this interaction increasing with the ongoing osteo-induction process. This last result was confirmed by the Duolink test performed in mouse tibial tissue, where the double positivity for the two proteins was evident along the side that separates the bone marrow from the bone, a site where likely MSCs and pre-OB are present.

This aspect leads us to hypothesize a possible co-operation between these two proteins inside these cells. This cooperation could consist in a carrier function of Fetuin-A for FGF23 molecule, given that Fetuin-A molecular structure makes it very suitable for a carrier role [25,44,45]. This hypothesis is supported also by our previous studies, where we described that FGF23 protein is always strictly linked to Fetuin-A also in bone, liver, and in the circulation but not in the renal tubule cells where FGF23 displays its canonical effects [23,24,25]. 

However, to better understand the possible nuclear mechanism(s) by which FGF23 might affect Fetuin-A production we performed a CHIP with FGF23 on MSCs cells at T0 and T5 followed by a PCR amplification with Fetuin-A promoter primers. The positivity of a band at the position of Fetuin-A promoter (189 bp) confirmed that FGF23 directly binds this DNA sequence. This result was completely overlapping that obtained in OS, where we already demonstrated the stimulatory effects of FGF23 on Fetuin-A production [23,24]. This is reinforced using two different primers for Fetuin-A promoter and of negative and positive controls. We also explored the effects of both FGF23 overexpression and addition on FGF23 and Fetuin A mRNA in M2-10B4 cells. The over-expression of FGF23 was followed by a consistent increase in both FGF23 and Fetuin-A. These results were quite similar to those previously described in OS [23,24]. At variance, the exogenous addition of FGF23 resulted in different changes of FGF23 and Fetuin-A, depending on the stage of maturation of M2-10B4 cells. In fact, both Fetuin A and FGF23 mRNAs, which are very low in M2-10B4 at T0, progressively increased after the addition of increasing concentrations of FGF23, up to 800 pg/mL, but starting to reduce at higher FGF23 levels; on the other hand at T5, when both mRNAs are already consistently increased before FGF23 addition, the stimulatory effect of FGF23 on their levels was evident only at the lowest concentration (400 pg/mL), followed by a reduction with higher FGF23 concentrations. Though these results are not easy to explain, it could be hypothesized that the increased basal production of FGF23 could compete with the exogenously added protein in the control of mRNA activation. In support of this interpretation, at T7, when the differentiation toward the OB phenotype is close to being completed and the production of FGF23 in basal conditions is maximized, the addition of FGF23 at any concentration was not only ineffective in any stimulation but even induced a marked reduction of both FGF23 and Fetuin-A mRNAs activation. These results, though supporting a role of both proteins in the maturation process of M2-10B4 towards OB, suggest that this role could be different depending on the stages of osteo-differentiation. To gain more confirmation on this point, we also explored the effects of exogenous FGF23 on surrogate markers of osteo-differentiation, namely BMP2 and RUNX2. The osteo-induction, without FGF23 addition, produced an increase of both BMP2 and RUNX2 in MSCs from T0 to T7. Then, a scalar FGF23 stimulation at T0 induced an increase for both proteins starting to be significant from 800 to 1600 pg/mL. At variance, at T5 the addition of 400 pg/mL of FGF23 stops inducing increase of both proteins. This apparently different behavior might represent a switch condition. Indeed, the subsequent addition of exogenous FGF23 at more advanced stages of osteo-differentiation were followed by an inhibition of the explored markers. These results might suggest a competitive mechanism on the osteo-differentiation process between the exogenously and endogenously produced FGF23. 

Our data might also help to explain the results from a study where co-culture of MSCs and OS induced an osteo-differentiation of the former cells with a concomitant increase of ALP and calcium deposition [46], suggesting that OS secretion of FGF23 might represent the differentiating factor. 

This could be an intriguing point, since chronic kidney disease patients, who are burdened by an overwhelming degree of vascular calcification, also have very high levels of FGF23, which could act as a promoter of MSC differentiation toward OB/OS cells [47,48]. 

HYD, one of the compounds used in the osteo-differentiation process, is well known to induce Fetuin-A promoter in the liver [49]. So, we have decided to rule out that HYD was the real culprit of Fetuin-A increase in our experiment. In fact, MSC differentiation towards the OB line was stimulated by all compound combinations of the medium, though to a different degree, with the maximal effect having been observed when all the reagents were added together. However, when FGF23 was silenced, these effects were almost completely suppressed, strongly suggesting that the osteo-induction process could work through the production of FGF23.

## 4. Material and Methods

### 4.1. Tissues

All efforts were made to minimize animal suffering and to use only the minimum number of animals necessary. All animals were housed on a 12-h light/dark cycle, allowed free access to food and water, and killed by decapitation after anesthesia induced by intraperitoneal injection of 370 mg/Kg of Chloral hydrate. All procedures involving animals were performed in accordance with the European legislation (European Communities Council Directive of 24 November 1986, 86/609/ EEC) and complied with the European Communities Parliament and Council Directive of 22 September 2010 (2010/63/EU) and with the Italian (D.L.vo n. 26/2014) and were reviewed and approved by the licensing and Ethical Committee of the IRCCS-AOU San Martino-IST National Cancer Research Institute, Genoa (Italy) protocol No. 371, and by the Italian Ministry of Health.

Bone tibial tissues of 6-week-old male balb/c mice (Charles River Italy, Inc., Lecco) were treated with decalcifying solution (HCl 37%/Formic acid/85% distilled water) for 6 h, fixated in paraformaldehyde 4%, and embedded in paraffin.

### 4.2. Human and Mouse Bone Marrow, Osteocytes (OS), and Podocytes (PODO) Cell Line Culture

Human L88/5 (kindly provided by Dr. Karin Thalmeier) and mouse M2-10B4 bone-derived MSCs (BMMSC) (ATCC^®^ CRL-1972^TM^, Sesto San Giovanni, MI, Italy) were cultivated in medium Roswell Park Memorial Institute (RPMI-1640) supplemented with 10% of fetal bovine serum (FBS) and 1% of penicillin/streptomycin (P/S) (all from Sigma–Aldrich, Milan, Italy). Since it is well recognized that MSCs are sensitive to the different types of protocols used, when cells reached 80% confluence, the transformation in OB was performed using the standardized osteogenic inducer reagent (Takara Bio. Inc., Kusatsu, Japan) following the instructions until different time points, namely T0–T15 [50,51]. 

Conditionally *immortalized* PODO SV1 (SV1) from transgenic *H-2Kb*-tsA58 mice (CLS, Eppelheim, Germany) were plated on collagen type IV (Sigma–Aldrich) at 33 °C with Dulbecco’s modified eagle medium (DMEM: F12) supplemented with 10% fetal calf serum, 1% P/S, 2 mM L-glutamine, and 20 U/mL recombinant mouse γ interferon (all from Sigma–Aldrich). Subsequently, PODO were thermo-shifted to 37 °C for differentiation for 7 days and grown with the complete medium without γ-interferon in pre-coated flasks. 

The MC3T3-E1 cell line was obtained from ATCC^®^ CRL-1972^TM^ and cultured in alpha minimum essential medium (α-MEM) (Invitrogen, Milan, Italy), supplemented with 10% FBS and 1% P/S in 75-cm^2^ flasks at a density of 400,000 cells. When cells reached 80% confluence, 50 µg/mL ascorbic acid (AA) and 3 mM glycerol 2-phosphate disodium salt hydrate (GP) (Sigma–Aldrich) were added and the medium was changed every 2 days. After 5 days of incubation with AA/GP, the cells were re-plated at a density of 7000 cells/cm^2^ and cultured with basal medium plus AA/GP and 10 uM All Trans Retinoic Acid (Sigma–Aldrich) to obtain the OS. Osteocytes were studied on the 4th day of treatment [52,53]. 

### 4.3. Primary MSCs Isolation and Culture

Mesenchymal cells from 3 bone marrow aspirates (bone marrow-derived MSCs, BMMSCs), 3 lipoaspirates (adipose-derived MSCs, ADMSCs), and 3 human cord blood units (cord blood-derived MSCs, CBMSCs) were obtained from healthy donors after informed consent. Human BMMSCs (Protocol: M. 36.F.CONS. REV. 2. of 20 January 2017), human ADMSCs (Protocol: M. 02.438.CONS. REV. 3. of 6 September 2016), and human CBMSCs (Protocol: P.01.CLT. M01 REV. of 11 July 2017) were all approved by our institution.

The BMMSC culture and isolation were performed as previously described [54]. Briefly, unprocessed bone marrow was directly seeded in alpha-MEM supplemented with 10% FBS at the concentration of 50,000 total nucleated cells/cm^2^ in 75-cm^2^ flasks. After 72 h, non-adherent cells were removed by washing with PBS with complete medium change. Medium changes were performed twice a week. On day 14 (±3), MSC at P_0_ were detached using TrypLE-Select (Gibco-Life Technologies, Carlsbad, CA, USA) and re-seeded in the same culture conditions at the concentration of 4000 MSCs/cm^2^. 

The ADMSC culture and isolation were performed as previously described [55]. Briefly, after lipoaspirate centrifugation, the lower-density solid phase was collected and treated with 0.075% of collagenase (Roche, Mannheim, Germany). The stromal vascular fraction was cultured in the previously described proliferation supporting medium, and after 48 h, fresh medium was added to primary cultures. Every 3 days cells were passaged to select only colonies of adherent cells. The CBMSC culture and isolation were also performed as previously described [56,57]. Briefly, CB nucleated cells were incubated for 20 min with RosetteSep MSCs enrichment cocktail for lineage-negative depletion of hematopoietic cell types (StemCell Technologies, Vancouver, Canada). The resulting cells were plated in proliferation supporting medium as above and monitored for MSC colony appearance in the following 2 weeks changing medium twice a week. The characterization of primary MSCs was verified by flow cytometry analysis of specific surface antigens and monitored by appropriate stains for adipocytes, bone, and chondrocytes after differentiation (Figure S2).

### 4.4. Alkaline Phosphatase (ALP) and Collagen Type I Stain 

Cells were seeded in 12-well plates and triplicates were studied per each time point. After fixation in cold acetone, the activity staining of ALP was evaluated using a commercial kit following the manufacturer’s instructions (tartrate-resistant acid phosphatase (TRACP) and ALP double-stain kit Takara Bio. Inc.). For evaluation of COLIαI, L88/5, and M2-10B4 cell lines were cultured on cover slips for 24 h, fixed in cold acetone for 5 min room temperature (RT), and permeabilized with 0.3% of Triton X-100/PBS (Sigma–Aldrich) for 30 min RT and finally incubated with 1% of bovine serum blocking solution for 30 min RT. Immunofluorescence (IF) was then performed with COLIαI- fluorescein isothiocyanate (FITC) for 1 h RT. For picrosirius red (to visualize collagen fiber), after fixation with methanol for 15 min RT, the cells were stained with a solution of Sirius Red 0.1% (Direct Red 80; Fulka, Sigma) in a picrosirius red solution (Sigma) for 1 h RT. Thereafter, the cells were washed in 0.01 N HCl (Sigma) for 2 min RT. 

### 4.5. mRNA Extraction and qRT-PCR

Cells were seeded in 12-well plates and 3 replicates were studied at each time point. Total RNA of M2-10B4 and L88/5 cell lines and primary BMMSC, ADMSC, and CBMSC were extracted by Trizol (Invitrogen, Milan, Italy), precipitated by chloroform and isopropanol, washed in ethanol 75%, treated with DNase (Invitrogen) and resuspended in nuclease free-water for spectrophotometric quantification. cDNA was prepared from 500 ng RNA using the iScript Select cDNA Synthesis Kit and oligo(dt)20 primers (Bio-Rad, Segrate, Milan, Italy). After assessment of primer specificity, the mRNA extracted was used to evaluate human FGF23 and Alpha-2-HS-Glycoprotein (AHSG) and mouse FGF23, AHSG, BGLAP, bone morphogenic protein 2(BMP2), RUNX2 and AHSG promoter. In order to verify the absence of genomic DNA in the samples, qRT-PCR was performed in triplicates also on the original RNA (minus-reverse transcriptase). Data were normalized against the expression of human Ribosomal Protein L4 (RPL4) and mouse RPL13. The qRT-PCR were run with iQ Sybr^®^ Green Supermix (Bio-Rad) on a MyIQ instrument (Bio-Rad), and data were analyzed by the IQ5 Bio-Rad Software. All primers information is given in Table S1. 

### 4.6. Immunohistochemistry (IHC) and IF

Immunohistochemistry (IHC) of dewaxed tibial tissues was performed with Rb polyclonal FGF23 [M-251] and monoclonal Alpha-2-HS-glycoprotein [2H2] for 1 h RT followed by broad spectrum horse-radish peroxidase HRP polymer conjugated. 

Primary BMMSC, ADMSC, CBMSC, human L88/5, and mouse M2-10B4 cell lines were cultured on cover slips for 24 h, fixed in cold acetone for 5 min RT, and permeabilized with 0.3% of Triton X-100/PBS (Sigma–Aldrich) for 30 min RT, and finally incubated with 1% of bovine serum blocking solution for 1 h RT. The immunofluorescence for human cells was performed with rabbit (Rb) polyclonal FGF23 and mouse (Ms) monoclonal Alpha-2-HS-glycoprotein [2H2], alternatively for mouse cells Rb polyclonal FGF23 [M-251] and Ms monoclonal Alpha-2-HS-glycoprotein [2H2] for 1 h RT were used. As secondary antibodies, Alexa Fluor 546 Goat anti-Rb immunoglobulin G (IgG), Alexa Fluor 488 Goat anti-Ms IgG, and Alexa Fluor 546 Goat anti-Ms IgG were incubated for 1h RT. Specificity of antibody (Ab) labelling and control negative was performed by IgG Isotype control Rb and IgG1 Isotype control Ms. The nuclei were stained with DAPI. Images were acquired by Zeiss AxioObserver microscope equipped with a high-resolution digital video camera (AxioCam, Zeiss) and Apotome system for structured illumination and recorded by AxioVision software 4.8. All antibody information is given in Table S2.

### 4.7. FGF23 and Fetuin-A Overexpression and Silencing

Twenty-thousand M2-10B4 cells/cm^2^ were transfected with 10 nM FGF23 mouse cDNA clone (open reading fragment with green fluorescent protein tagged C-terminal) (OriGene Technologies Inc.MD, USA) using Lipofectamine 2000 (Invitrogen) as a transfecting agent. Mouse FGF23 cDNA clone: Vector: pCMV6_AC-GFP, Tag: C-terminal TurboGFP, Sequence Data: (Genbank: NM_022657.3) ORF Size:756 bp, restriction sites: Sgfl-Mlul.

For FGF23 silencing 20,000 M2-10B4 cells/cm^2^ were transfected with 10 nM siRNA duplexes using Lipofectamine 2000 (Invitrogen) as the transfecting agent. Three commercially available siRNAs complementary to FGF23 mRNA were used (Sigma–Aldrich). First sequence forward 5′ GCU AUC ACC UAC AGA UCC A 3′ reverse primer 5′ UGG AUC UGU AGG UGA UAG C 3′, second sequence forward primer 5′ CCA UAG GGA UGG UCA UGU A 3′ reverse primer 5′ UAC AUG ACC AUC CCU AUG G 3′, third sequence forward primer 5′ CUC GAA GGU UCC UUU GUA U 3, reverse primer 5′ AUA CAA AGG AAC CUU CGA G 3′. As control, only Lipofectamine 2000 was applied. Transfection efficiency was determinate by a fluorently-tagged siRNA (Alexa-Fluor488; Amersham, PerkinElmer, Waltham, MA).

### 4.8. Protein Interaction

Duolink (Sigma–Aldrich) in situ experiment was performed to detect Fetuin-A/FGF23 interactions in M2-10B4, L88/5, OS mouse cell lines, in the primary ADMSC, BMMSC, CBMSC and in the tibial mouse tissue following the manufacturer’s instructions. Cells and tissue were fixed in PFA for 10 min, blocked for 30 min (Sigma–Aldrich), and stained with anti-FGF23 and anti-Alpha-2-HS-glycoprotein (all antibodies information in Table S2) overnight (o/n) at 4 °C. A second incubation with PLUS/MINUS PLA probe was performed and after ligation and amplification (both from Sigma–Aldrich), we visualized the interaction by IF. As control negative, we chose to omit one of the two antibodies alternatively.

### 4.9. Chromatine Immunoprecipitation (CHIP) and Semi-Quantitative PCR

Chromatine immunoprecipitation of 10^6^ cells of OS at 4 days and M2-10B4 were performed using the standard protocol (Thermofisher, USA) with antibodies rabbit anti-FGF23 and anti-normal rabbit IgG as control negative (Thermofisher). Polymerase chain reaction was performed on 10% on DNA called input on CHIP with FGF23 and on control negative and control positive. All antibody information is provided in Table S2.

Primers tested on Fetuin-A promoter are designed on the Mus musculus strain 129 AHSG gene complete cds (Gene Bank: AH005595.2 NCBI). One pair of them was designed on a core promoter region of AHSG (MPRM17140 NM_013465) containing canonical cis-regulation elements including the TATA box and the transcription start site (TSS). Such a region was also deeply investigated by bioinformatic tools and a literature review [58] and verified by Blast. The second pair of primers were designed in a region flanking the minimal promoter containing regulatory elements [59] (all information given in Figure S1). Amplification conditions: PCR buffer with 2.5 mM MgCl2, 200 nM each primer and fast Start Taq in the presence of 200 nM dNTPs (Roche). Amplification step step 1: 6’ at 95 °C, step 2: 35’’ at 95 °C, step 3: 35’’at 57, 5–60 °C, step 4: 50’’at 72 °C, step 5: from step 2 (×12), step 6: 35’’at 95 °C, step 7: 35’’ at 57 °C, step 8: 50’’ at 72 °C, step 9: from step 6 (×23), step 10: 9’at 72 °C, step 11: 4 °C. The presence of PCR products (279 bp and 189 bp) were resolved with loading dye on 2 % agarose gels containing 5 µl ethidium bromide in 0.5 x tris borate ethylenediaminetetraacetic acid (EDTA) (TBE) buffer alongside a low molecular weight DNA ladder (Invitrogen) and photographed under UV light using a GelDoc system (Bio-Rad).

### 4.10. Enzyme-Linked Immunosorbent (ELISA) Tests

The FGF23 levels in the cultured cell supernatants were evaluated by ELISA Kit (KAINOS laboratories, Japan). The minimal detectable concentration is 3 pg/mL. The FGF23 levels of MSCs supernatant were compared to OS (control positive), PODO (control negative), and cells culture media. Replicate background measurements were subtracted to all 450 nm measures. To normalize, Janus Green Whole-Cell Stain (Thermofisher) was added for 5 min. Careful washing was followed by addition of elution buffer and absorbance was read at 630 nm. The resulting A450–A630 values were then normalized to the A615 values to account for differences in numbers of cells.

### 4.11. Statistical Analyses

Experiments were conducted on 3 replicates per each condition and time point. For real-time RT-PCR, relative RNA abundance was determined using the comparative Ct method [60]. Fold change error bars, calculated automatically by the IQ5 Bio-Rad Software, represent the standard deviation (σ) of the fold change (FC), according to the following formula: σFC = FC × ln2 × sqrt(σx 2/nx + σy 2/ny). 

Data were expressed as mean ± standard deviation (SD), and Student’s *t*-test and Mann–Whitney test, when necessary, were applied to analyze the differences among groups. In all statistical analyses, the significance was set for *p*-values < 0.05. Statistical analyses were performed using the software SPSS^®^ version 20 (IBM, Armonk, USA).

## 5. Conclusions

In conclusion, our study suggests that FGF23 is produced also by MSCs cells, though not released. Fibroblast growth factor 23 stimulates Fetuin-A production in MSCs as in OS, and this stimulation being mediated by a direct interaction of FGF23 with Fetuin-A promoter.

Fibroblast growth factor 23 seems to drive the osteo-differentiation process of MSCs towards the OB line. The addition of FGF23 to the medium at an early stage of differentiation strongly stimulates this process, suggesting that high serum levels of this protein might play a role in the MSC/OB trans-differentiation also in in vivo conditions, such as in CKD patients with possible implications on mineral metabolism and then on the CV system.

## Supplementary Materials

It can be found at http://www.mdpi.com/1422-0067/20/4/915/s1.

## Abbreviations

MSCs	Mesenchymal stem cells
OB	Osteoblasts
FGF	Fibroblast growth factor
OS	Osteocytes
POD	Podocytes
HYD	Hydrocortisone
COLIαI	Collagen type i
ALP	Alkaline phosphatase
Rb	Rabbit
Hu	Human
BMMSCs	Bone marrows mesenchymal stem cells
ADMSCs	Adipose derived mesenchymal stem cells
CBMSCs	Cord blood mesenchymal cells
Ms	Mouse
AHSG/Fetuin-A	Alpha 2-hs glycoprotein
GP	Glycerol phosphate
AA	Ascorbic acid
CVD	Cardio vascular disease
MBD	Mineral bone disorder
CKD	Chronic kidney disease
