# Supplementary material for: FGF23 and Fetuin-A Interaction and Mesenchymal Osteogenic Transformation
Source: Int J Mol Sci. 2019 Feb 20;20(4):915. doi: 10.3390/ijms20040915 (PMC6412477; doi:10.3390/ijms20040915)
Supplement: Supplementary file 1 [file ijms-20-00915-s001.pdf]

Supplemetary data

Table S1: Primers information

GENE	NCBI	FORWORD PRIMERS 5' → 3'	REVERSE PRIMERS 5' → 3'
Human FGF23	NM_020638.2	CCTTGTGCCTCTCCTCTTTATC	GATTTCCTCTTCCCTACACCTTC
Human AHSG	NM_001622.2	GCAGCTCTGGTGGCTATAGA	CTTCGACAGCATGCTCCTTC
Mouse FGF23	AF263536	GATCCCCACCTCAGTTCTCA	CCGGATAGGCTCTAGCAGTG
Mouse AHSG	NM_001276450	CACCGAACTTACCACGACCT	ATGTCCTGTCTGCCAAAACC
Mouse BGLAP	NM_007541.3	GCAGAACAGACAAGTCCCAC	ACCTTATTGCCCTCCTGCTT
Mouse BMP2	NM_007553.3	CCCCAAGACACAGTTCCCTA	GAGACCGCAGTCCGTCTAAG
Mouse RUNX2	NM_001271631.1	TGGCTTGGGTTTCAGGTTAG	GGTTTCTTAGGGTCTTGGAGTG
Human RPL4	NM_000968.3	CGAGCACCACGCAAGAAGATCCA	AATGGTGTTCCGGCGCATGGT
Mouse RPL13	NM_016738.5	TACTGAAGCCCCACTTCCAC	CGGACCTGGGTGTGGTATCT
Mouse Fetuin-A promoter primer A		TCAGATAAATTAGGCCCTCTGC	TGTTTGAATCCCTGAAGAAGA
Mouse Fetuin-A promoter primer B		CCAAGTCGTCGCCTACAAAG	GCCAAGAAGCCTTCACACAA

Table S2: Antibodies information

Antibody	Species Clone	Source	Catalogue	Final Concentration	Usages
**Primary antibody**					
FGF23	Rabbit polyclonal	Abcam	ab98000	10 µg/ml	IF (human), Duolink
Alpha-2-HS glycoprotein [2H2]	Mouse monoclonal	Abcam	ab128120	10 µg/ml	IF, Duolink (human&mouse)
FGF23 [M-251]	Rabbit polyclonal	Santa Cruz	sc50291	2 µg/ml	IF (mouse)
Collagen Type I-FITC		Sigma	C4361	1 mg/ml	IF
DAPI		Sigma	D9542	1 µg/ml	IF
RNA polymerase II		Thermo Scientific	186243	10 µl	CHIP
Normal rabbit IgG		Thermo Scientific	1862244	1 µl	CHIP
**Secondary antibody**					
Alexa Fluor 546	Goat anti-Rabbit IgG	Invitrogen	A11035	14 µg/ml	IF
Alexa Fluor 488	Goat anti-Mouse IgG	Invitrogen	A11029	14 µg/ml	IF
Alexa Fluor 546	Goat anti-Mouse IgG	Invitrogen	A11030	14 µg/ml	IF
**Negative control**					
IgG isotype control	Rabbit	Invitrogen	PA5-23090	3 µg/ml	IF
IgG1 isotype control	Mouse	Invitrogen	MA5-14453	3 µg/ml	IF

Table S3: qRT-PCR of FGF23 and Fetuin-A in the adipogenesis and condrogenesis

FOLD CHANGE	BONE MARROW T0	BONE MARROW T10	ADIPOCYTES T21	CONDROCYTES T21
FETUIN-A	**1**	**2.801** (**P=0.001)	**3.481** (***P=0.0002)	**1.230** (P=0.666)
FGF23	**1**	**5.409** (**P=0.001)	**0.386** (P=0.346)	**0.767** (P=0.716)

Figure S1

PRIMER A (279 bp)

TSS **TATA BOX**

4561 TACGCAATTCCTTCGGCGGGCTCTG**TCAGATAAATTAGGCCCTCTGC**CCCTCTATTGGTC
4621 TAGCTCTCCAAGCTGATTATCCGGGCTGCTCCTGACATTTGCCCATTTTCCAGGGCCTCT
4681 CTGGAGCAACC**ATG**AAGTCCCTGGTCTTGCTCCTTTGTTTTGCTCAGCTCTGGGGCTGCC
4741 AATCCGCTCCACAAGGTACAGGACTGGGTTTTAGAGAATTGGCTTGTGATGATCCAGAAG
4801 CAGAGCAAGTAGCTTTGTTGGCCGTGGACTACCTCAATAATCA**TCTTCTTCAGGGATTCA**
4861 **AACA**GGTCTTGAATCAGATCGACAAAGTCAAGGTGTGGTCTCGGGTAAGTGAGCCTACCA

PRIMER B (189 bp)

3421 GGTTCCCATTCAGGTCTGTACATGTGCGTGTGGCTGGAAGCAAATGCAAAATGAGCCCTG
3481 CTACCACTGTCTCTGCCCTGTCACCCTCCCTCTCTACCCACAA**CCAAGTCGTCGCCTACA**
3541 **AAG**TTGGTGCCATTTAGATTTCCATCAGAAGTTCTTTCTTCTCTTCTTTTGTAACAAGTA
3601 TTCCCCCCCCCCCCCCCGAAGCAATGTTAAAAACCACAGGACAAATCCATCCAAATGTAA
3661 CGGTGACTTCGCGGTATTTGAAGTATGGTCT**TTGTGTGAAGGCTTCTTGGC**TCCCGGTCC

Figure S1. Sequence of primers A and B on Fetuin-A core promoter sequence. TATA box, and TSS sites (transcription start sites) localization on Primer A.

Figure S2

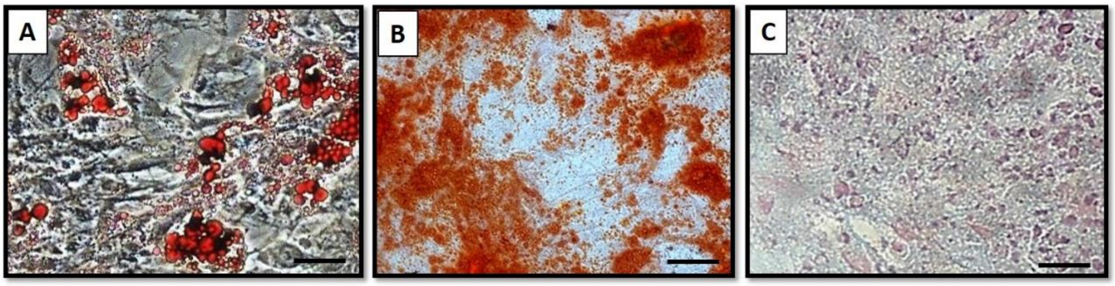

D

Antigens	BMMSC	CBMSC	ADMSC
CD90	98.0	97.5	99.9
CD73	99.3	100.0	96.3
CD105	93.6	97.6	99.1
CD45	0.2	0.4	0.8
CD34	0.1	0.0	0.3
CD14	0.2	0.5	0.1

Figure S2. Representative images of MSC differentiated into Oil Red O-positive adipocytes (A), Alizarin Red S-positive osteoblasts (B) and Alcian blue-positive chondrocytes (C) are shown. Scale bars respectively 25, 200, 50 µm. Table D showed percentages of positive cells for MSCs (CD90, CD73, CD105) and hematopoietic (CD45, CD34, CD14) specific surface antigens specified for each MSC type used in this study.

Figure S3

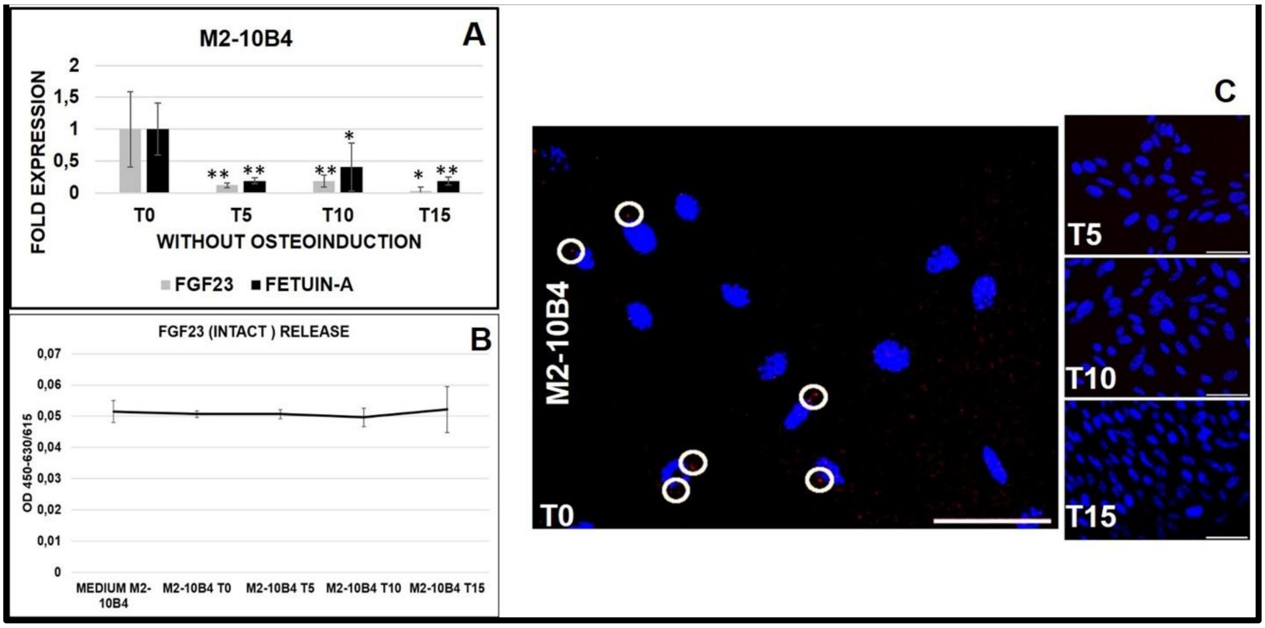

Figure S4

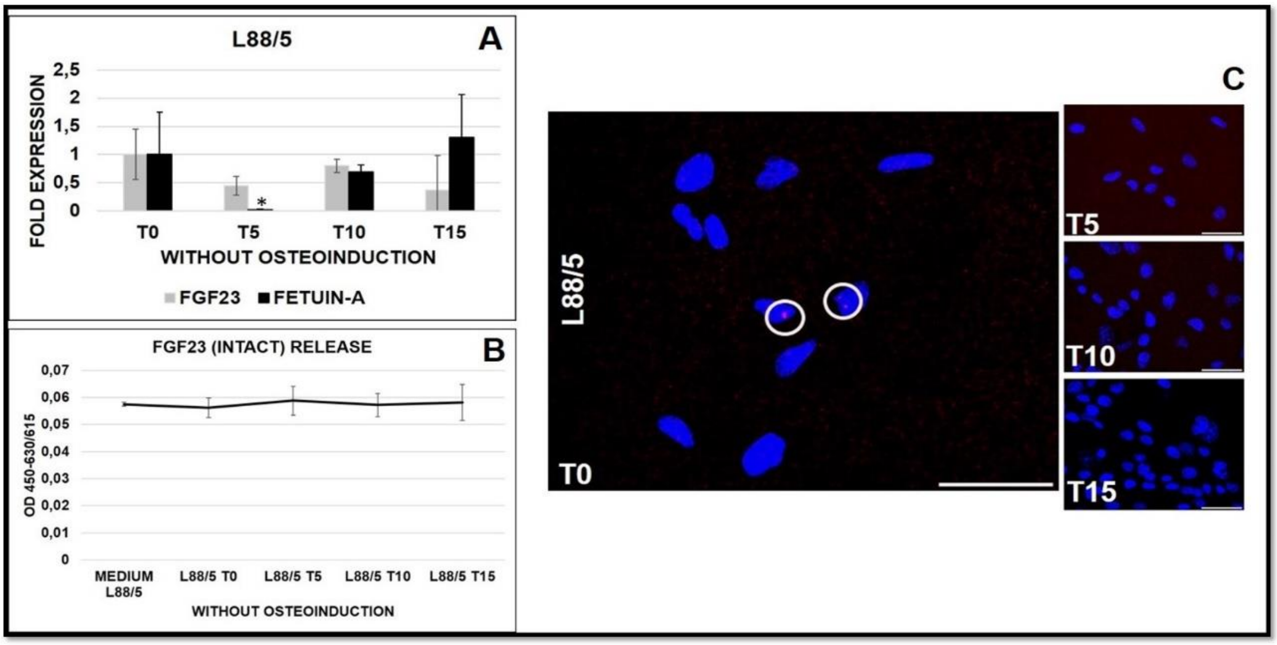

Figure S3-S4: qRT PCR of FGF23 and Fetuin-A mRNA expression in M2-10B4 (S3A) and L88/5 bone marrow cells (S4A) from T0 toT15 days from the osteogenic induction. Asterisks indicate significant differences versus M2-10B4 and L88/5 T0: *=p<0.05, **=p<0.01. Cell localization of Fetuin-A/FGF23 interaction detected by Duolink in situ experiment in M2-10B4 (S3C) and L88/5 (S4C) from T0 to 15 days from the osteogenic induction. Scale Bars: 50 µm.

Figure S5

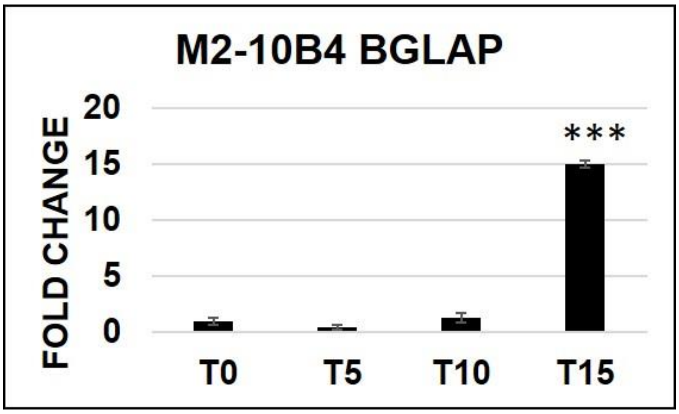

Figure S5: qRT PCR of BGlap mRNA expression in M2-10B4. Asterisks indicate significant differences versus M2-10B4 T0 (ctrl): ***=p<0.001.

Figure S6

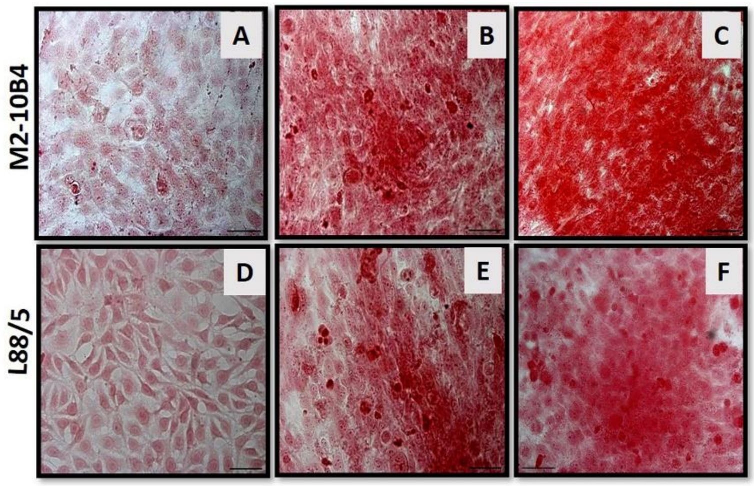

Figure S6: Picrosirius red staining on mouse M2-10B4 (A-C) and human L88/5 (D-F) bone marrow cells from T0 to T10 days from the osteogenic induction. Scale Bars: 50µm.
